# Quality of life and psychological concerns of adult patients with disorders of sex development

**DOI:** 10.1192/j.eurpsy.2023.2334

**Published:** 2023-07-19

**Authors:** N. Bouayed Abdelmoula, B. Abdelmoula, A. Karra

**Affiliations:** Genomics of Signalopathies at the service of Medicine, Medical University of Sfax, Sfax, Tunisia

## Abstract

**Introduction:**

Disorders of sex development (DSD) are characterized by an atypical development of chromosomal, gonadal, and anatomic gender. DSD are divided to three classes with male normal, female normal and abnormal karyotypes and have an incidence at birth of approximately 1 in 5000. DSD patients are exposed during their long-term follow-up to severe psychological stress.

**Objectives:**

The aim of our study was to determine the behavioral concerns of adults with DSD and to identify the major factors that may influence their emotional and psychological well-being.

**Methods:**

We explored, through our genetic counselling reports at the medical University of Sfax, the quality of life and the psychological concerns of all adults patients assessed for DSD with and without chromosomal and/or molecular genetic abnormalities. We also assessed their need of psychological support.

**Results:**

During the last two decades of our genetic counselling experience, 46 adult patients (age superior to 18 years) were selected for this study. The analysis of data revealed that the major psychological concern in our DSD patients was related to their reproductive capacity. In contrast, they have poor subjective norms of communicating sexual and reproductive issues with their partners. Patients who presented non-corrected ambiguous genitalia were in the majority anxious and depressed with a constant feeling of social shame.

**Conclusions:**

Sex development plays a fundamental role in determining the physical attributes of the body, the structure of the brain, behavioral tendencies, and the self-concept. The clinical and social approaches of DSD conditions in our society need to be improved. Genetic and psychological counselling should thorough a deep medical education regarding reproductive and sexual health in each particular case of DSD. We emphasis, that early genetic diagnosis, involvement of patients and families in a patient-centered decision-making process, and consideration of long-term health-related quality-of-life outcomes should be considered in DSD.

**Disclosure of Interest:**

None Declared

